# Prenylated Flavonoids from Roots of *Glycyrrhiza uralensis* Induce Differentiation of B16-F10 Melanoma Cells

**DOI:** 10.3390/ijms19082422

**Published:** 2018-08-16

**Authors:** Yunfeng Zheng, Huaiyou Wang, Min Yang, Guoping Peng, Tina Ting Xia Dong, Miranda Li Xu, Karl Wah Keung Tsim

**Affiliations:** 1 School of Pharmacy, Nanjing University of Chinese Medicine, Nanjing 210023, China; zyunfeng88@126.com (Y.Z.); yangmingnj90@163.com (M.Y.); guopingpeng@sohu.com (G.P.); 2 Division of Life Science and Center for Chinese Medicine, The Hong Kong University of Science and Technology, Hong Kong 999077, China; hyw@ust.hk (H.W.); botina@ust.hk (T.T.X.D.); lxuae@connect.ust.hk (M.L.X.); 3 Shenzhen Key Laboratory of Edible and Medicinal Bioresources, The Hong Kong University of Science and Technology Shenzhen Research Institute, Hi-Tech Park, Shenzhen 518057, China

**Keywords:** *Glycyrrhiza uralensis*, prenylated flavonoids, antiproliferation, differentiation, melanoma cell

## Abstract

Roots of *Glycyrrhiza uralensis* have been used as herbal medicine and natural sweetener. By activity-guided phytochemical investigation of the extracts from *G. uralensis* root, ten flavonoids, namely GF-1–GF-10, of which five were prenylated flavonoids, were found to show antiproliferative effects in melanoma B16-F10 cells. Three of the prenylated flavonoids, namely GF-1, GF-4 and GF-9, significantly induced the differentiation of B16-F10 cells; the inductions included increase of tyrosinase activity, tyrosinase protein, and melanin content. In GF-1 and GF-9 induced melanoma differentiation, the phosphorylation of p38 MAPK (mitogen activated potein kinase) was identified; while GF-4 could trigger the phosphorylation of PI3K/AKT (phosphatidylinositol 3-kinase/Protein Kinase B) signaling. However, application of GF-6 to the melanoma cells did not induce differentiation; but which promoted cell apoptotic signaling, i.e., increase levels of cleaved-PRAP, cleaved-caspase 3, and cleaved-caspase 9. These results suggested that different types of prenylated flavonoids from *G. uralensis* might have potential anticancer effects against melanoma cells by acting through different signaling pathways.

## 1. Introduction

The roots of *Glycyrrhiza uralensis* Fisch (GlycyrrhizaeUralensis Radix; GUR), named as licorice or gancao, have been extensively used as a herbal drug in both Eastern and Western countries [1]. Clinical studies have shown GUR is highly effective in the treatment of respiratory, gastrointestinal, cardiovascular and genitourinary conditions [2]. According to traditional Chinese medicine (TCM) theory, GUR is described as a beneficial herb to enhance therapeutic effects; meanwhile, this herb is being employed commonly to detoxify potential adverse effects in many herbal mixtures during clinical application. Phytochemical studies revealed that triterpene saponins and flavonoid glycosides were two major active substances in GUR [3]. In the extractive of GUR, the nonpolar fraction of GUR is rich in various types of prenylated flavonoids, e.g., flavones, isoflavones, flavanones, chalcones, and coumestans [4]. These flavonoids are being considered to be active ingredients of GUR, and indeed which have been commonly used in food industries. In recent pharmacological studies, GUR flavonoids have been proposed to have anti-oxidation [5], anti-inflammatory [6], antiproliferative, and cytotoxic effects in various cells [7]. Despite the aforementioned proposed actions, the roles of GUR prenylated flavonoids in cancer cells have not been extensively investigated.

Malignant melanoma is a highly aggressive and invasive skin cancer with high metastatic potential and extraordinary resistance to cytotoxic agents [8]. The occurrence of melanoma is related to different factors, e.g., sun exposure, fair pigmentation, and genetic mutation [9]. In recent years, the incidence of melanoma is rapidly increasing throughout the world, especially in America and Europe [10]. Although drug therapy for this cancer has been developed rapidly, e.g., immunotherapies with PD-1 inhibition drugs (ipilimumab and nivolumab) and T-cell checkpoint blockade therapies [11,12], the usage of herbal products is still one of the alternative approaches for melanoma cancer treatment.

Considering the pathogenesis and clinical treatment of melanoma, the recent targets in cancer therapy is focusing on discovery of natural products that are able to suppress cancer cell proliferation and promote cell differentiation [13,14]. Here, the CH_2_Cl_2_ extract of GUR (GUR_CH2Cl2_) was shown to exhibited potent effect in antiproliferation and inducing differentiation of cultured melanoma B16-F10 cells: these effects were significant higher than that deriving from water extract (GUR_water_) and ethanol extract of GUR (GUR_EtOH_). To explore the possible underlying mechanism for anticancer effect of GUR, ten flavonoids (GF1-GF10) of which five were prenylated flavonoids, were isolated. The roles of these flavonoids in inducing the differentiation of cultures B16-F10 cells were illustrated, and subsequently the signaling cascades, triggered by various flavonoids, were revealed and compared.

## 2. Results 

### 2.1. G. uralensis Extracts in Proliferation and Differentiation of Melanoma Cells

**Figure 1:**
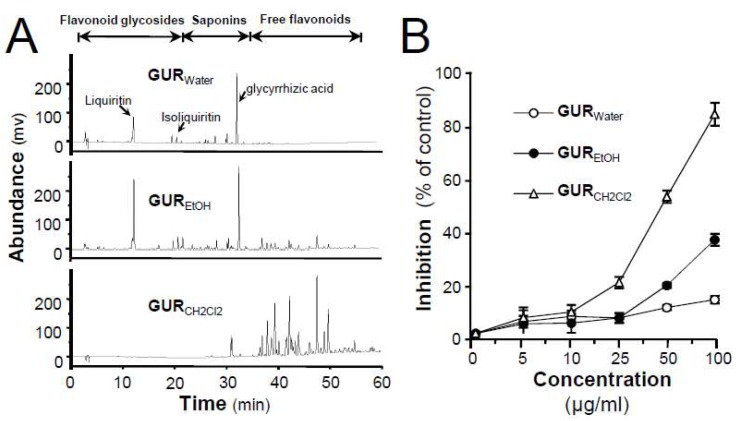

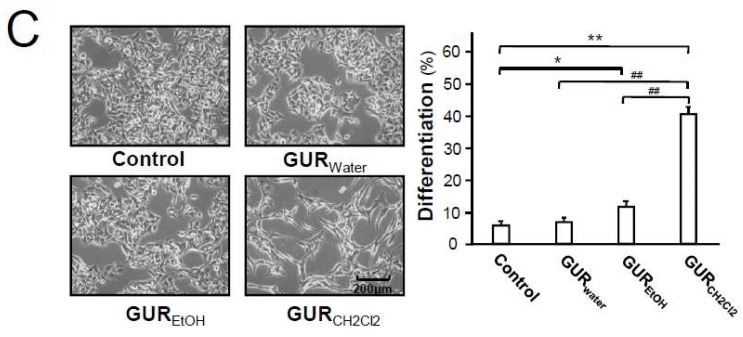
Effects of different extracts of *G. uralensis* root in antiproliferation and differentiation-inducing activities in B16-F10 cells. (**A**) HPLC chromatograms of different extracts, all at 1 mg/mL, from GUR, i.e., GU_water_ (water extract of GUR), GU_EtOH_ (EtOH extract of GUR), and GU_CH2Cl2_ (CH_2_Cl_2_ extract of GUR). (**B**) Melanoma B16-F10 cells were treated with different extracts of GUR or with medium of 0.1% DMSO (dimethylsulphoxide) for 48 h, after which cells were counted under MTT (3-(4,5-dimethyl-2-thiazolyl)-2,5-diphenyl-2-H-tetrazolium bromide) assay. (**C**) Melanoma B16-F10 cells were treated with GU_water_, GU_EtOH_, and GU_CH2Cl2_ (50 μg/mL, respectively) for 48 h (left panel). At least 150 cells were counted for cells with dendrite longer than 3× cellular body, as differentiated cells (right panel). Data are expressed as percentage of control, in means ± SEM of 3 independent experiments. * *p* < 0.05, ** *p* < 0.01, compared with control; ## *p* < 0.01, compared with other extracts.

Different extractives of GUR, i.e., GU_water_, GU_EtOH_, and GU_CH2Cl2_, were subjected to HPLC analyses. As shown in HPLC chromatograms (Figure 1A), saponins and flavonoid glycosides were the major constituents in both GU_water_ (water extract) and GU_EtOH_ (ethanol extract). Besides, a small amount of free flavonoids could be detected in GU_EtOH_. In comparison with GU_water_ and GU_EtOH_, GU_CH2Cl2_ (dichloromethane extract) exhibited a comparable enrichment of free flavonoids. To a certain extent, difference in chemicals might cause possible difference in their biological capacities.

To investigate the effect of *G. uralensis* extracts, cultured B16-F10 melanoma cells were treated with different extracts at indicated concentrations for 48 h. As shown in Figure 1B, GU_CH2Cl2_ inhibited the proliferation of B16-F10 cells with an IC_50_ value of 48.7 ± 2.5 μg/mL, whereas GU_water_ and GU_EtOH_ exhibited little effects (IC_50_ > 100 μg/mL). In addition to the differentiation induction, as shown in Figure 1C, the GU_CH2Cl2_-treated (50 μg/mL) cells showed typical dendrite-like cellular protrusions, as compared with control cells. Although GU_EtOH_ had a certain degree of differentiation-inducing activity, as compared with the control group, its effect was far lower than that of GU_CH2Cl2_. Over 40% cells were induced to differentiate under the treatment of GU_CH2Cl2_ (Figure 1C). Similar to antiproliferative activity, GU_water_ showed little morphological changes towards cultured B16-F10 cells. These results suggested that free flavonoid content in the extract could be responsible for differentiation and antiproliferation of B16-F10 cells.

### 2.2. Structure Identification of Isolated Compounds

To explore active compounds in GUR extracts corresponding for activitieson cell differentiation, phytochemical investigation of GU_CH2Cl2_ was performed, and there after 10 compounds were isolated. Their structures and types were elucidated on the basic of ESI-Q-TOF/MS and ^1^H and ^13^C NMR and by comparison with the literatures. The ^13^C NMR data were presented in Table 1, and the MS and ^1^H NMR data of each compound were available in the supplementary information.

**Figure 2:**
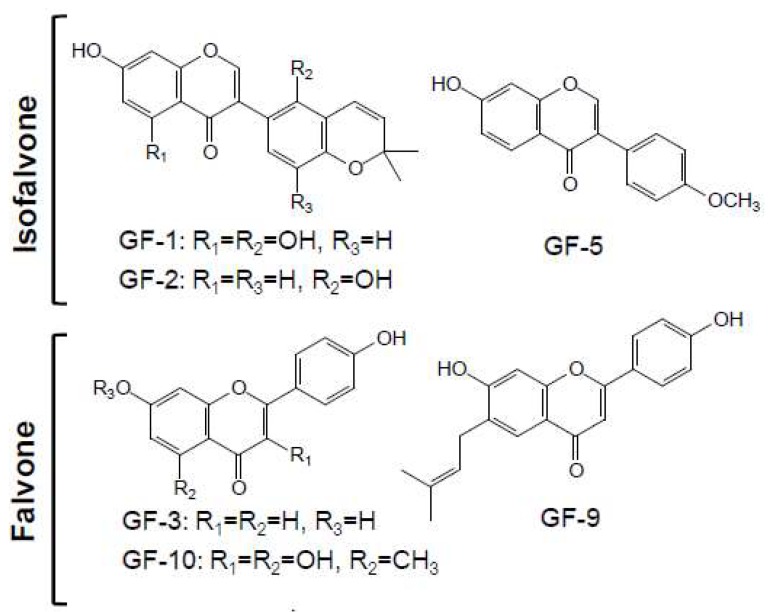

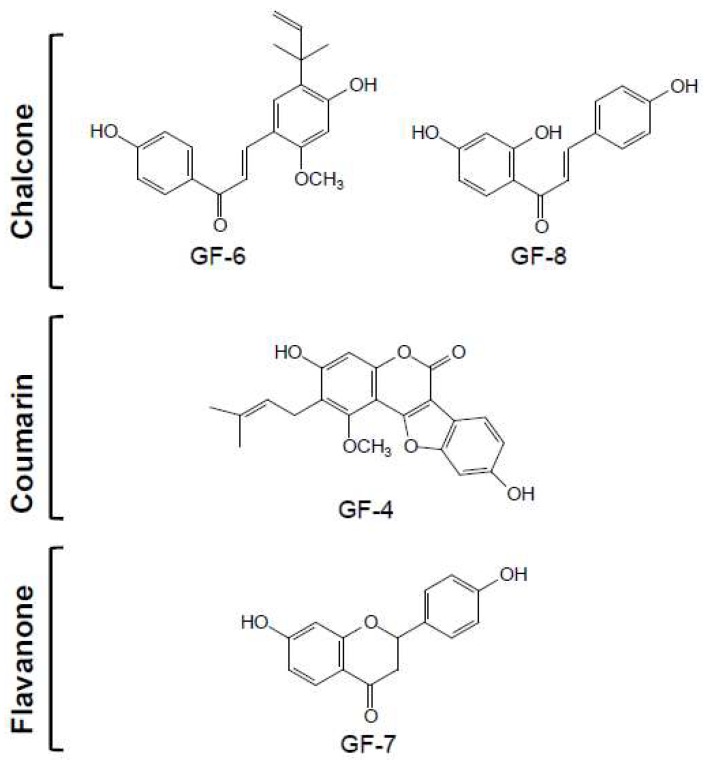
Structures of flavonoids isolated from CH_2_Cl_2_ extract of *G. uralensis* root.

As shown in Figure 2, all isolated compounds were identified as free flavonoids, including three isoflavones, three flavones, two chalcones, one coumarin, and one flavanone, namely GF-1–GF-10. Compounds GF-1, 2, and 5 were isoflavones, identified as licoisoflavone B, glabrone, and formononetin, respectively. Compounds GF-3, 9, and 10 were classified as flavones and elucidated successively as 7, 4′-dihydroxyflavone, licoflavone, and kumatakenin A, respectively. With the data of NMR and MS, two chalcones, GF-6 and GF-8 were established as licochalcone A and isoliquiritigenin, respectively. GF-4 was identified as a coumarin namely as neoglycyrol, which could be regarded as an isoflavone derivative with methoxyl, hydroxyl, and prenyl groups. GF-7 was identified as a common flavanone, namely as liquiritigenin. The HPLC of isolated flavonoids and dichloromethane extract of *G. uralensis* were available in the supplementary information. The purities of these ten flavonoids were tested to be higher than 95% by HPLC-UV area normalization method.

In recent years, flavonoids, especially the prenylated ones, have attracted extensive attention because of its resulting radicals can enhance their biological activities [15]. In this study, several flavonoids, including GF-1, 2, 4, 6, and 9, were found to contain a substituent of prenyl moiety or dimethylpyran (formation from prenyl) in their structures.

### 2.3. Isolated Flavonoidsin Proliferation and Differentiation of Melanoma Cells 

**Figure 3:**
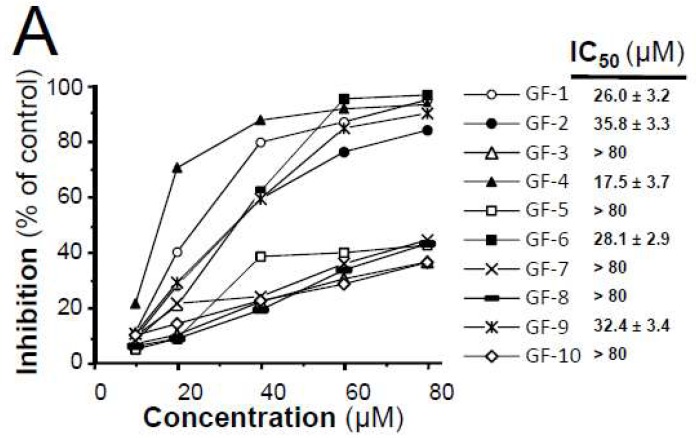

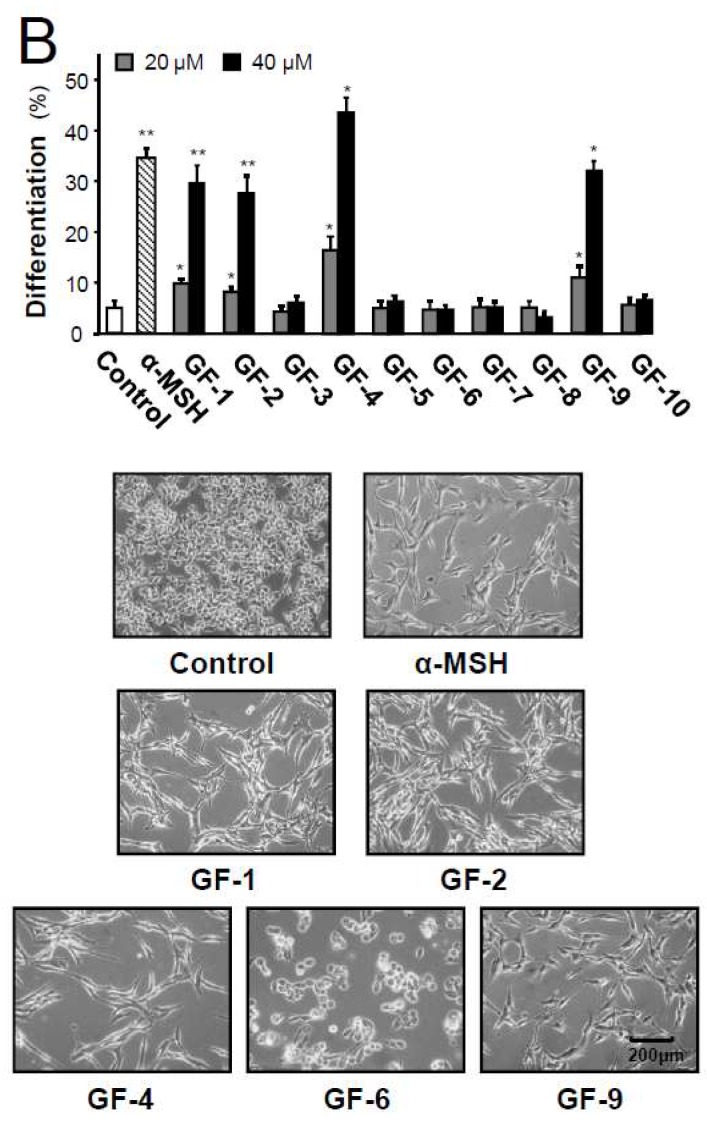
Antiproliferation and differentiation activity of *G. uralensis* flavonoids on B16-F10 cells. (**A**) Melanoma B16-F10 cells were treated with 10 flavonoids (GF-1–GF-10) at different concentrations or with medium (0.1% DMSO) for 48 h, after which the cells were assayed under MTT assay. IC_50_ value was calculated using SPSS statistics software. (**B**) Melanoma B16-F10 cells were treated with 10 flavonoids (GF-1–GF-10) at 20 or 40 μM, positive drug (α-MSH) at 20 nM or medium (0.1% DMSO) for 48 h. At least 150 cells were counted for dendrite longer than 3× cell body, as differentiated cell (upper panel). Data are expressed as percentage of control, as means ± SEM (*n* = 3) * *p* < 0.05, ** *p* < 0.01, compared with control. Morphological of B16-F10 cells treated with selected flavonoids at 40 μM (lower panel).

The isolated flavonoids were tested for their antiproliferative capacity using MTT bioassay on cultured B16-F10 cells (Figure 3A). In cell inhibition, the most active component was GF-4 (neoglycyrol), with an IC_50_ of 17.5 μM, while compounds GF-1, 2, 6, and 9 showed good activities in the range of 26.0–35.8 μM. The other nonprenyl flavonoids, GF-3, 5, 7, 8, and 10, possessed low activities of cell inhibition. Thus, the prenyl could be an important group for inhibition activity of licorice flavonoids in cancer cell growth.

In order to evaluate the differentiating effect of isolated flavonoids, cultured B16-F10 cells were treated with α-MSH (positive drug) or these compounds at two concentrations for 48 h. Accordingly, typical dendrite-like cellular protrusions of cells, induced by GF-1, 2, 4, and 9, were similar with those induced by 20 nM α-melanocyte-stimulating hormone(α-MSH) (Figure 3B) [16]. Surprisingly, GF-6 had no such phenotype induction regardless of its role in cell inhibition (Figure 3B). After comparison of structures of above compounds, the opening of central C-ring in GF-6 could be a possible domain in inducing differentiation in cultured B16-F10 cells.

### 2.4. Isolated Flavonoids in Melanogenesis

**Figure 4:**
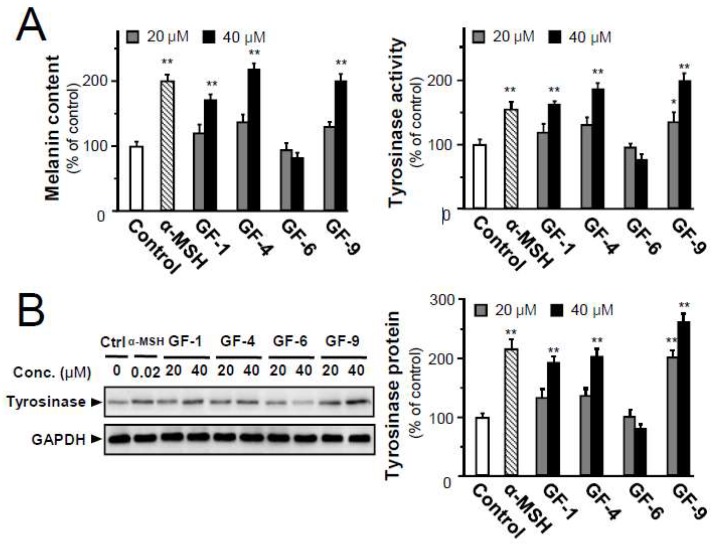
The *G. uralensis* flavonoids induce the amounts of melanin content, tyrosinase activity, and tyrosinase expression of B16-F10 melanoma cells. (**A**) B16-F10 cells were treated with indicated concentrations of selected flavonoids (GF-1, 4, 6, and 9) or with α-MSH at 20 nM for 48 h. The amounts of melanin (left panel) and tyrosinase activity (right panel) were determined. (**B**) The level of tyrosinase protein (~91 kDa) in the melanoma cells was measured by Western blot (left panel). The amount of tyrosinase was qualified against GAPDH (~37 kDa) (right panel). Data are expressed as percentage of control, in means ± SEM (*n* = 3). * *p* < 0.05, ** *p* < 0.01, compared with control.

Melanoma cell differentiation is generally accepted to be related to inhibition of cell proliferation, dendritic-like morphology, increased tyrosinase activity, and melanin production [17]. To validate the implications of morphological observations, the amounts of intracellular melanin content, tyrosinase activity and protein were evaluated in B16-F10 cells with the treatment of α-MSH or selected prenylated flavonoids, i.e., GF-1, 4, 6, and 9. The application of α-MSH (positive drug) at 20nM, GF-1, 4, and 9 at 40 μM significantly increased the melanin amounts by about 2-fold, as compared to the control (Figure 4A). However, GF-6 showed no effect. Except for GF-6, the prenylated flavoniods could induce tyrosinase activity in a dose-dependent manner in cultured B16-F10 cells (Figure 4A). The level of tyrosinase protein in the melanoma cells was measured by Western blot analysis. The expression of tyrosinase protein was enhanced to about 2-fold in GF-1, 4, and 9 treated cells (Figure 4B), demonstrating that the melanogenesis promotion was partly mediated by increased tyrosinase in melanoma cells.

**Figure 5:**
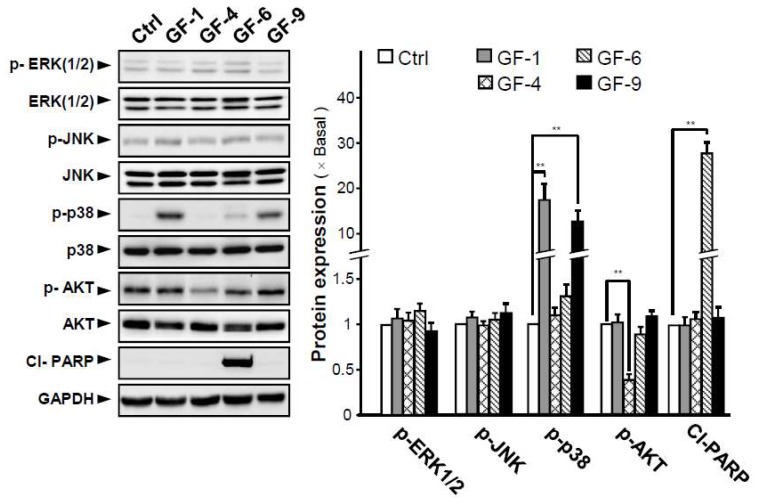
The *G. uralensis* flavoniods induce signaling of ERK (1/2), JNK, p38, AKT, and PARP. B16-F10 cells were treated with selected flavonoids (GF-1, 4, 6,and 9) at concentration of 40 μM or with medium (0.1% DMSO) for 48 h. Cell lysates were analyzed by Western blotting with the indicated antibodies (left panel). Photographs of the chemiluminescent detection of the blots, which were representative of three independent experiments, are shown (right panel). The relative abundance of each band to their own GAPDH was quantified, and the control levels were set at 1.0. Data are expressed as percentage of control, in means ± SEM (*n* = 3). ** *p* < 0.01, compared with control.

To explore potential mechanism of the four prenylated flavonoids (GF-1, 4, 6, and 9) on antiproliferation and differentiation-inducing activities in B16 cells, the activations of MAPKs (ERK 1/2, JNK, and p38), AKT (Protein Kinase B), and PARP (poly ADP-ribose polymerase) signaling pathwayswere probed in the treated B16-F10 cells. These signaling pathways have been identified as important regulators of cell proliferation, differentiation, and tumor development of the malignant phenotype of tumor [18]. The levels of phospho-ERK1/2 (p-ERK1/2) and phospho-JNK (p-JNK) remained unchanged after treatment with the four prenylated flavonoids (Figure 5). However, the level of phospho-p38 (p-p38) was significantly induced by treatment of GF-1 and GF-9, with ~16- and ~13-fold of increase, in respect to the control. In the phosphorylation of p-AKT, only GF-4 showed a reduction (Figure 5). In the cultures, GF-6 showed cell inhibition, but not in cell differentiation, and did not cause phosphorylation of p-ERK1/2, p-JNK, p38, and p-AKT. In contrast, GF-6 application in B16-F10 cells induced the phosphorylation of Cl-PRAP by ~30-fold (Figure 5). Thus, the four tested prenylated flavonoids showed distinction in inducing the signaling cascades, i.e., p-ERK1/2 and p-JNK triggered by GF-1 and GF-9, p-AKT triggered by GF-4, and Cl-PARP triggered by GF-9.

**Figure 6:**
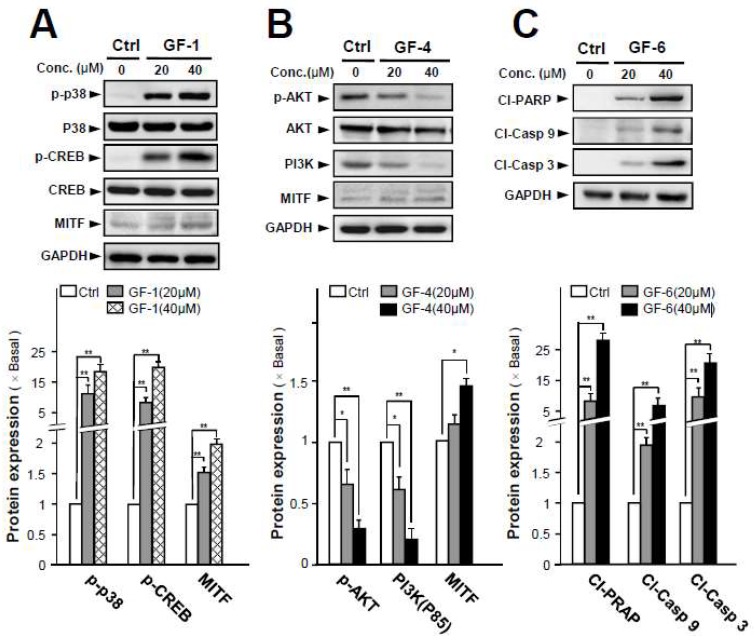
*G. uralensis* flavonoids induce differentiation or apoptosis via different signaling pathways in B16-F10 cells. B16-F10 cells were treated with various concentrations of selected flavonoids at concentration of 20 and 40 μM for 48 h, after which protein expression in total cells were assayed by Western blot analysis. (**A**) GF-1 and GF-9 induced phosphorylations of p 38 (~38 kDa) and p-CREB (~43 kDa), as well as expression of and MITF (~60 kDa). (**B**) GF-4 induced protein expression of p-AKT (~60 kDa), PI3K(~85 kDa), and MITF (~60 kDa). (**C**) GF-6 induced expression of Cl-PARP(~89 kDa), Cl-Casp 9(~37 kDa), and Cl-Casp 3(~19 kDa). The relative abundance of each band to their own GAPDH was quantified, and the control levels were set at 1.0 (lower panel). Data are expressed as percentage of control, in means ± SEM (*n* = 3).* *p* < 0.05, ** *p* < 0.01, compared with control.

The phosphorylation of p38 could lead to activation of cAMP response element binding protein (CREB) and induction of microphthalmia associated transcription factor (MITF) expression [19]. MITF is a known downstream effector that is required for melanoblast survival, as well as a key regulator to induce genes associated with the differentiation, e.g., tyrosinase [20]. Here, GF-1 and GF-9 markedly increased the phosphorylations of p-p38 and p-CREB, as well as promoting the expression of MITF in dose-dependent manners (Figure 6A).

The PI3K/AKT signaling pathway is another crucial pathway involved in melanoma cell differentiation. Application of GF-4 down-regulated the phosphorylation of p-AKT and PI3K in cultured B16-F10 cells, and this suppression was in a dose-dependent manner (Figure 6B). Glycogen synthase kinase 3 β, a direct downstream target of AKT, phosphorylates MITF and regulates expression of tyrosinase [21]. Therefore, the protein expression of MITF was determined under the treatment of GF-4 in cultured B16-F10 cells. As expected, the level of MITF was induced by 50% after the treatment of GF-4 (Figure 6B). The GF-6-induced Cl-PRAP expression suggested that apoptosis could be involved in the inhibition of B16-F10 cells, triggered by GF-6. In line with an increase of Cl-PRAP, the levels of cleaved caspase-3 and cleaved caspase-9 were markedly induced by treatment of GF-6: the maximal induction over 5-fold and 15-fold could be revealed in cleaved caspase-3 and cleaved caspase-9, respectively (Figure 6C). The increase of apoptotic biomarkers strongly suggested the possible anticancer function of GF-6.

## 3. Discussion 

It has been estimated that 30–40% of cancers can be prevented by dietary or lifestyle condition [22]. Some anticancer drugs currently used in clinics, such as paclitaxel and camptothecin, are derived from natural products. Thus, the search for natural products having anticancer activity represents an interesting area, probably due to its diversity and distinct action mechanism. Flavonoids, commonly found in foods or herbal medicines, are a group of compounds with potential antitumor activities. According to their variations of backbone and substitution, flavonoids can be classified into different subclasses, providing an extremely diverse range of derivatives. Among them, prenylated flavonoids have attracted considerable attention due to their promising and diverse bioactivities [15]. For example, the prenylated substituted chalcones were shown to be cytotoxic in MCF-7, HT-29, and A-2780 cancer cells, whereas the conversion of a chalcone to a favanone resulted in reduced antiproliferative activity [23]. In addition, a prenylated flavonoid, artonol A, exhibited cytotoxic activity against human lung cancer cells [24]. Thus, the substitution in flavonoid structure might possess a profound influence on their antiproliferative effect on cancer cells. As shown here, the proliferation of B16-F10 cells could be significantly inhibited by CH_2_Cl_2_ extract of GUR. From this extract, ten flavonoids were isolated, of which five flavonoids with a prenylated group, i.e., GF-1, 2, 4, 6, and 9, displayed effectively cytotoxic activity: these results suggested that the prenyl group could be an indispensable moiety for licorice flavonoids to inhibit B16-F10 cell growth. Considering the structure-activity relationship, the free isoprene flavonoids, e.g., deprenylated compound of GF-9 and kumatakenin B, are showing low antiproliferative effect on B16-F10 cells.

The differentiation process represents a crucial point in progression of many types of cancer. A potential cancer treatment is to promote cancer cell differentiation, and thereafter, to reduce its proliferative capacity: this approach is a novel therapeutic method aiming to modify tumor cells to a slower rate of proliferation, and thus which leads to the loss of its earlier neoplastic attributes [25,26]. In melanoma, the differentiation status of cells could be monitored by melanogenesis, which resulted in the conversion of l-tyrosine to l-dopa by tyrosinase [27]. Indeed, the levels of melanin and tyrosinase are being considered as indicators of differentiated melanoma cells. Our current results supported the contrary action of differentiation against proliferation in melanoma. Here, we have shown the prenylated flavonoids GF-1, 4, and 9 inhibited B16-F10 cell growth, and subsequently which induced cell differentiation. The differentiation, induced by the prenylated flavonoids, was illustrated by morphological change and increase of melanin and tyrosinase. Having a distinction here, GF-6 (licochalcone A) showed inhibitory effect only on cell growth but not differentiation. The GF-6-inhibited cell growth was revealed to be mediated by its apoptotic role. In line to this observation, licochalcone A was shown to activate apoptotic processes in both in vitro and in vivo [28]. Thus, different backbone structure of flavonoids, as revealed here for *G.uralensis* flavonoids, might have distinct mechanisms to inhibit growth of tumor cells.

Numerous studies have shown that the MAPKs and AKT signaling pathways could play role in survival, proliferation, and differentiation of melanoma [18]. The MAPKs include three well characterized sub-families, i.e., extra cellular signal-regulated kinases -1/2(ERK 1/2), c-jun N-terminal or stress-activated protein kinases (JNK), and p38 mitogen activated protein kinase (p38 MAPK). The activations of ERK (1/2) and JNK were shown to relate with suppression of melanogenesis [20]. Activation of p38 MAPK signaling could upregulate melanin synthesis and induce differentiation by promoting expressions of MITF and tyrosinase [19]. In addition, the activation of AKT could inhibit melanogenesis and differentiation of melanoma [29]. These different lines of evidence support the antiproliferation and differentiation-inducing effects of natural products on melanoma, and the regulation of MAPKs and AKT signaling could be one of key mediators. For example, 5, 7-dimethoxycoumarin, from *Citrus limon* L., showed potential antigrowth and differentiation-inducing effect on melanoma cell, involving Ras/Raf/MEK/ERK pathway [30]. Lupeol, a lupine-type triterpene from Danelion root, showed significant inhibition on melanoma cell growth both in vitro and in vivo: the effect was trigged by its induction on cell differentiation [31]. Ye [27] reported that three sesquiterpenes, isolated from the largehead atractylode rhizome, induced melanoma cell differentiation and inhibited cell migration through inactivating the signals of Ras/ERK MAPK (for AT-I and AT-II) and PI3K/AKT. Here, we isolated and identified several prenylated flavonoids including isoflavone, flavone, chalcone, and coumarin. Interestingly, prenylatedflavone (GF-1) and prenylatedisoflavone (GF-9) could induce activation of p38 MAPK pathway, while prenylated coumarin (GF-4) showed inhibition on PI3K/AKT signaling. The prenylated chalcone GF-6 did not affect cell differentiation, but which induced melanoma cell apoptosis with activation of proteins in caspase-mediated signaling pathway. This result was in good agreement with recent literature [32]. However, a more detailed mechanism by which the prenylated flavonoids in melanoma cell differentiation and cell proliferation are waiting for further elucidation.

## 4. Material and Methods

### 4.1. Plant Material

The roots of *G. uralensis* (GUR) were collected in Minqin County, Gansu province, China in September 2016. Voucher specimen (No. 20160919) was stored in a dry and dark room and deposited at Jiangsu Collaborative Innovation Center of Chinese Medicinal Resources Industrialization (Nanjing University of Chinese Medicine). The authentication of GUR was carried out by Prof. Qinan Wu from Nanjing University of Chinese Medicine, Nanjing, China. 

### 4.2. Extraction and Isolation 

#### 4.2.1. Phytochemical Extractions of *G. uralensis* Roots

The water extract of GUR (GUR_water_) was prepared by boiling GUR crude materials (100 g of fine powder) with water (1000 mL, 1 h) at 100 °C, extracted twice. Likewise, 100 g GUR powder was extracted with ethanol (1000 mL, 1 h) at 80 °C, extracted twice to obtain the ethanol extract (GU_EtOH_). To prepare GUR dichloromethane extract (GUR_CH2Cl2_), 100 g of GUR powder was boiled at 50 °C (1000 mL, 1 h) with CH_2_Cl_2_, extracted twice. The fractions were concentrated in vacuumand then freeze-dried to obtain loose fine powder.

HPLC chromatograms of different GUR extracts (GUR_water_, GUR_EtOH_, and GUR_CH2Cl2_) were carried out using an Agilent 1100 HPLC system (Agilent Technologies, Santa Clara, CA, USA). An Agilent RP C_18_ column (250 × 4.6 mm id, 5 μm) was used. Samples were separated using a gradient mobile phase consisting of 0.2% (*v*/*v)* formic acid water (A) and acetonitrile (B). The gradient conditions were: 18–25% B at 0–10 min, 25–45% B at 10–30 min, 45–70% B at 30–45 min, and 70% B at 45–60 min. The flow rate was set at 1.0 mL/min. The detection wavelength was 254 nm. The sample concentration was 1 mg/mL, and injection volume was 10 μL.

#### 4.2.2. Isolation and Identification of Flavonoids

GUR (dry weight, 5 kg) were exhaustively extracted two times with dichloromethane (30 L × 2, each extraction for 1 h). The extractives were combined and concentrated in vacuo. The residue (a total of about 105 g) was then separated to three fractions (Fr. A–C) by normal-phase silica gel CC (800 g, 200–300 mesh Silica) using a step-wise gradient elution of CH_2_Cl_2_-MeOH (100:0–80:20, *v*/*v*).Fr. A (about 6.5g) applied to middle pressure liquid chromatography (MPLC, BUCHI Chromatography B-688, Switzerland) on ODS column with a gradient solution of MeOH-H_2_O (60:40–70:30, *v*/*v*) as elution to give GF-1 (43 mg) and GF-2 (18 mg). Fr. B (about 31.6 g) was subjected to MPLC silica gel using a stepwise gradient elution of CH_2_Cl_2_-MeOH (95:5–90:10, *v*/*v*) to provide the two sub-fractions Fr. B1 and Fr. B2. Fr. B1 was subsequently purified by MPLC chromatography over RP-18 silica gel column (400 g, 25–50 μm, 4.5 × 50 cm) eluted with MeOH-H_2_O (70:30) to afford GF-3 (55 mg), GF-4 (102 mg) and GF-5 (31 mg). Fr. B2 was subjected to MPLC with a ODS column (400 g, 25–50 μm, 4.5×50 cm) to give GF-6 (26 mg) and GF-7 (34 mg). Fr. C (about 9.4 g) was applied to MPLC chromatography on ODS column with a gradient solution of MeOH-H_2_O (60:40–70:30, *v*/*v*) as elution to afford GF-8 (23 mg), GF-9 (42 mg), and GF-10 (67 mg). The purified compounds were characterized by LC-MS and NMR analyses. The MS spectra were recorded on an Agilent 1200 HPLC/Q-TOF mass spectrometer instrument (Agilent) in positive ion mode. The specimens were dissolved in DMSO-d_6_ (dimethylsulphoxide), and ^1^H NMR and ^13^C NMR were assayed with ASR-500 or ASR-300 NMR spectrometer, nuclear magnetic resonance, TMS was used as an internal standard.

### 4.3. Cell Culture

The melanoma B16-F10 cell line was obtained from American Type Culture Collection (ATCC, Manassas, Virginia, VA, USA). The cells were maintained in Dulbecco’s modified Eagle’s medium containing 10% fetal bovine serum, 100 units/mL penicillin and 100 μg/mL streptomycin in a humidified CO_2_ (5%) incubator at 37 °C. All reagents for cell cultures were purchased from Invitrogen (Carlsbad, California, CA, USA). 

### 4.4. Antiproliferative Activity Assay

The antiproliferative activities of GUR extracts and flavonoids were investigated using the MTT assay [14]. Firstly, B16-F10 cells were cultured in 96-well plates at approximately 7.5 × 10^3^ cells per well and incubated for 12 h. Then cells were treated with different concentrations of GUR extracts (5, 10, 25, 50, and 100 μg/mL) or GUR flavonoids (10, 20, 40, 60, and 80 μM). After incubation of 48 h, MTT solution (5 mg/mL in PBS) was added to each well, and cells were incubated at 37 °C for 4 h. Subsequently, 150 μL DMSO was added to each well, and the plate was put on a shaker for 5 min, the absorbance were measured at 570 nm using a fluorescence plate reader (Thermo scientific, Waltham, MA, USA). Inhibition percentage (%) was calculated according to the following formula: Inhibition percentage of cell viability (%) = [1−(OD treated well/OD control well)] × 100%. The IC_50_ value was calculated using the SPSS v. 22.0 Statistics Software (IBM Corp., Armonk, New York, NY, USA).

### 4.5. Morphological and Differentiation Analysis

B16-F10 cellswere cultured in 35-mm dishes (2 × 10^5^ cells/well) and treatedwith different concentration of GUR extracts (0, 50 μg/mL), its flavonoids(0, 20, 40 μM), or α-MSH (20 nM) for 48 h.Afterthe treatment, the morphological changes were observed under a microscope (Olympus, Japan). Differentiation and mature phenotypes were characterized by formation of dendrite-like cellular protrusions. Differentiation ratio (%) was expressed as the percentage of cells with cytoplasmic extension longer than three cellular bodies in relation tothe total number of cells [33,34].

### 4.6. Measurement of Tyrosinase Activity

Measurement of tyrosinase activity was performed according to a previously published method with slight modifications [27]. In brief, cells were cultured in 6-wells/plate at a density of 2 × 10^5^ cells/well, and treated with different concentrations of GUR flavonoids (0, 20, and 40 μM, respectively) or α-MSH (20 nM) for 48 h. After the treatment, the cells were washed with ice-cold PBS twice, lysed at 4 °C for 30 min in RIPA lysis buffer (150 mMNaCl, 1 mM EDTA, 1 mM EGTA, 50 mM Tris–Cl, 0.35% *w*/*v* sodium-deoxycholate, 1 mM phenylmethylsulfonyl fluoride, 1% *v*/*v* NP-40, 1 mM NaF, 1 mM Na_3_VO_4_, pH 7.4) containing a protease-inhibitor cocktail. Each lysate was centrifuged at 15,000× *g* for 30 min. Then, 50 μL of supernatant was added to 100 μL of 0.1% L-DOPA in PBS (pH = 6.8) in a well on a 96-well plate. After 2 h of incubation at 37 °C, the absorbance at 475 nm was measured. Meanwhile, the protein concentration of each lysate was determined by Bio-Rad protein assay. Tyrosinase activity was normalized with protein amount.

### 4.7. Determination of Melanin Content

Cellular melanin content was measured as described previously with a slight modification [14]. B16-F10 cells (2 × 10^5^ cells) were placed in 6-well/plate and treated with different concentrations of GUR flavonoids (0, 20, and 40 μM, respectively) or α-MSH (20 nM) to determinate melanin content of B16-F10 cells. After incubation for 48 h, the cells were washed twice with PBS, then cells were collected after digestion with 0.05% Trypsin-EDTA for cell counting. Then 1 × 10^5^ cells per well were dissolved in 150 μL of 1 M NaOH containing 10% DMSO for 1 h at 80 °C. The lysate was centrifuged at 16,000× *g* for 20min. Then, 100 μL supernatant was transferred to 96-well plates. The absorbance of each well was measured at 490 nm by fluorescence plate reader.

### 4.8. Western Blot Analysis 

Western blot was performed as previously described [35]. B16-F10 Cells cultured in 100-mm dishes were treated with the required GUR flavonoids concentration of 0, 20, and 40 μM or α-MSH (20 nM) for 48 h. Then, cells were collected and proteins were extracted with RIPA lysis buffer containing a protease-inhibitor cocktail. The total protein concentrations were measured using the Bradford method, and the normalized protein samples were added to 4× sample buffer, then boiled and denatured. Equal amounts of proteins were separated by SDS-PAGE and then transferred to nitrocellulose membranes. The membranes were blocked and then probed with indicated primary antibodies, respectively, with anti-GAPDH (1:5000), anti-tyrosinase (1:200), anti-ERK1/2 (1:2500), anti-p-ERK1/2 (1:1000), anti-JNK (1:2500), anti-p-JNK (1:1000), anti-p38 (1:2500), anti-p-p38 (1:1000), anti-AKT (1:2500), anti-p-AKT (1:1000), anti-cleaved-PARP (1:1000), anti-P-CREB (1:1000), anti-CREB (1:1000), anti-MITF (1:200), anti-PI3K(P85) (1:1000), anti-caspase 3 (1:2500), anti-caspase 9 (1:2500), anti-cleaved-caspase 3 (1:1000), anti-cleaved-caspase 9 (1:1000), at 4 °C overnight. Anti-tyrosinase and anti-MITF antibodies were purchased from Santa Cruz. Biotech (Santa Cruz, California, CA, USA), others were from Cell Signaling Technology (Cell Signaling, Danvers, Massachusetts, MA, USA). All antibodies were diluted with 5% BSA in TBST buffer. The blots were rinsed and then incubated with secondary antibodies (anti-mouse antibody or anti-rabbit antibody, 1:5000, Cell Signaling Technology). Reactive bands were visualized using ECL (Thermo Fisher Scientific; Waltham, MA, USA) and then calibrated by Chemidoc Imaging System (Bio-Rad; Hercules, California, CA, USA). 

### 4.9. Statistical Analysis

The significant difference among groups was statistically performed by one-way analysis of variance (ANOVA) combined with Tukey’stest by SPSS v. 22.0 program (IBM Corp., Armonk, New York, NY, USA). Probability value less than 0.05 (*p* < 0.05) was considered to be statistically significant. Data are expressed as the means±standard error of mean (SEM) of at least three independent experiments. 

## 5. Conclusions

Here, we provided different lines of evidence that the dichloromethane extract of *G. uralensis* roots has potential antiproliferation and differentiation-inducing activities in cultured B16-F10 melanoma cells. Prenylated flavoniods were isolated and demonstrated to be the active constituents within the extract of *G. uralensis* roots. These prenylated flavonoids have different backbone structures, and thus each of them has distinct activity in inhibiting cancer cell growth. The present results provided the molecular basis for using nonpolar extract of *G. uralensis* roots, and the identified bioactive prenylated flavonoids could be further developed for the prevention and/or treatment of melanoma cancer.

## Figures and Tables

**Table 1:** Table of ^13^C NMR Spectroscopic data of 10 flavonoid compounds in DMSO-d6.

	GF-1	GF-2	GF-3	GF-4	GF-5	GF-6	GF-7	GF-8	GF-9	GF-10
C-1						113.5		125.7		
C-2	155.7	155.1	162.5	157.9	153.0	158.2	78.9	131.1	162.2	155.8
C-3	120.5	122.1	104.4	102.7	124.2	99.9	43.1	115.8	104.4	137.7
C-4	180.6	176.0	176.2	158.6	174.5	159.6	190.0	160.3	176.2	178.0
C-4a	104.6	116.2	16.1	100.2	127.2		113.5		115.8	105.1
C-5	161.8	127.2	126.4	154.3	116.6	126.5	128.3	115.8	124.5	160.9
C-6	98.8	115.3	114.7	120.1	115.0	127.7	110.5	131.1	127.2	97.6
C-7	164.1	162.8	160.6	160.0	162.5		164.6		160.3	165.0
C-8	93.6	102.0	102.4	99.7	102.0		102.5		101.7	92.2
C-8a	157.7	157.5	157.3	153.4	158.9		163.1		155.6	160.2
C-α						117.8		144.2		
C-β						138.7		117.4		
C-γ						187.3		191.5		
C-1′	111.2	112.7	121.7	114.7	123.1	129.6	129.3	112.9	121.9	120.4
C-2′	151.2	151.3	128.0	156.6	130.0	130.6	128.2	165.8	127.9	130.1
C-3′	109.7	110.1	115.8	99.0	113.5	115.2	115.1	102.5	115.8	115.6
C-4′	153.6	153.5	162.4	157.4	157.4	161.6	157.6	165.4	160.5	156.2
C-5′	107.4	107.6	115.8	114.5	113.5	115.2	115.1	108.0	115.8	115.6
C-6′	131.3	130.9	128.0	120.9	130.0	130.6	128.2	132.7	127.9	130.1
C-1″				22.5		39.5			27.5	
C-2″	75.4	75.4		122.9		147.5			121.8	
C-3″	128.8	128.8		131.4		109.9			132.3	
C-4″	116.9	117.0		18.2		26.9			25.5	
C-5″	27.4	27.5		25.9		26.9			17.6	
C-6″	27.4	27.5								
2-OCH_3_						55.4				
3-OCH_3_										56.0
4′-OCH_3_					55.1					
5-OCH_3_				62.8						
7-OCH_3_										59.6

## Supplementary Materials

Supplementary materials can be found at http://www.mdpi.com/1422-0067/19/8/2422/s1.
