# Supplementary material for: Prenylated Flavonoids from Roots of Glycyrrhiza uralensis Induce Differentiation of B16-F10 Melanoma Cells
Source: Int J Mol Sci. 2018 Aug 16;19(8):2422. doi: 10.3390/ijms19082422 (PMC6122034; doi:10.3390/ijms19082422)
Supplement: Supplementary file 1 [file ijms-19-02422-s001.pdf]

Supporting information

Prenylated flavonoids from roots of *Glycyrrhiza uralensis* induce differentiation of B16-F10 melanoma cells

Yunfeng Zheng [a], Huaiyou Wang [b,c], Min Yang [a], Guoping Peng [a], Tina T.X. Dong [b,c], Miranda L. Xu [b,c], Karl W.K. Tsim [b,c] *

Affiliation

[a] School of Pharmacy, Nanjing University of Chinese Medicine, Nanjing, China

[b] Division of Life Science and Center for Chinese Medicine, The Hong Kong University of Science and Technology, Hong Kong, China;

[c] Shenzhen Key Laboratory of Edible and Medicinal Bioresources, Shenzhen Research Institute, Hi-Tech Park, Shenzhen 518000, China

*To whom correspondence should be addressed: Prof. Karl W. K. Tsim, Division of Life Science, The Hong Kong University of Science and Technology, Clear Water Bay Road, Kowloon, Hong Kong, China; Tel: (852) 2358-7332; Fax: (852) 2358-1559; Email: botsim@ust.hk

Contents

Part 1. The MS and ^{1}H NMR data of 10 flavonoid compounds

Part 2. HPLC of dichloromethane extract of *G. uralensis* and isolated flavonoids

Part 1. The MS and ^{1}H NMR data of 10 flavonoid compounds

GF-1: faint yellow needle crystal; HR-ESI/MS: *m/z* 353.1024 $[M+H]^+$; molecular formula: $C_{20}H_{16}O_6$; ^{1}H NMR (DMSO-d$_6$, δ, ppm): 1.38 (6H, s, 5"-H and 6"-H), 5.68(1H, d, *J*=10.0, 3"-H), 6.23(1H, d, *J*=1.9, 6-H), 6.33(1H, d, *J*=8.3, 5'-H), 6.40(1H, d，*J*=1.9, 8-H), 6.67(1H, d, *J*=10.0, 4"-H), 6.89(1H, d, *J*=8.3, 6'-H), 8.18(1H, s, 2-H), 8.85(1H, s, 2'-OH), 10.83(1H, s, 7-OH), 12.85(1H, s, 5-OH). ^{1}H and ^{13}C NMR data were identical with the literature values of Licoisoflavone B [1].

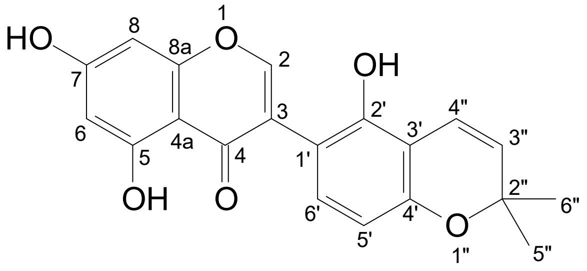

Licoisoflavone B

GF-2: faint yellow needle crystal; HR-ESI/MS: *m/z* 337.1071 $[M+H]^+$; molecular formula: $C_{20}H_{16}O_5$; ^{1}H NMR (DMSO-d$_6$, δ, ppm): 1.38 (6H, s, 5"-H and 6"-H), 5.68 (1H, d, *J*=10.0, 3"-H), 6.34 (1H, d, *J*=8.3, 5'-H), 6.68 (1H, d, *J*=10.0, 4"-H), 6.91 (1H, d, *J*=1.9, 8-H), 6.93 (1H, d, *J*=8.3, 6'-H), 6.97 (1H, dd, *J*=8.8, 1.9, 6-H), 7.98 (1H, d, *J*=8.8, 5-H), 8.25 (1H, s, 2-H), 9.11 (1H, s, 2'-OH), 10.85 (1H, s, 7-OH). ^{1}H and ^{13}C NMR data were identical with the literature values of glabrone [2].

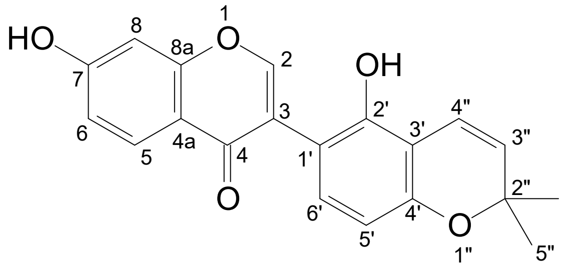

Glabrone

GF-3: yellow needle crystal; HR-ESI/MS: *m/z* 353.1012 $[M+H]^+$; molecular formula: $C_{20}H_{16}O_6$; ^{1}H NMR (DMSO-d$_6$, δ, ppm): 6.72 (1H, s, H-3), 7.87 (1H, *J*=9.0, H-5)，7.91 (2H, m, H-2', 6'), 6.97 (1H, d, *J* = 2.5 , H-8) , 6.93 (2H, m, H-3', 5'), 6.89 (1H, m, H-6), 10.73 (1H, br, 7-OH)，10.26 (1H, br, 4'-OH). ^{1}H and ^{13}C NMR data were in agreement with the literature values of 7, 4'-dihydroxyflavone [3].

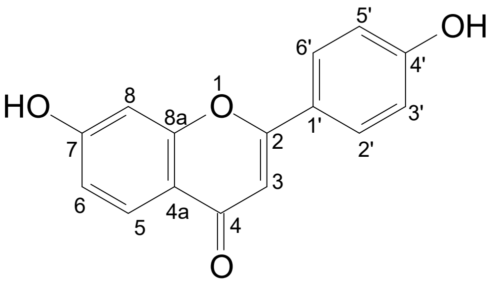

7, 4'-dihydroxyflavone

GF-4: faint yellow needle crystal; HR-ESI/MS: *m/z* 367.1187 $[M+H]^+$; molecular formula: $C_{21}H_{18}O_6$; ^1H NMR (DMSO-d_6, δ, ppm): 1.76 (6H, s, 4''-H and 5''-H), 3.32 (2H, d, *J* =6.8, 1''-H), 3.90 (3H, s, 5-OCH_3), 5.19 (1H, m, 2''-H), 6.77 (1H, s, 8-H), 6.95 (1H, dd, *J*=2.0, 8.4, 5'-H), 7.17(1H, d, *J*=1.6, 3'-H), 7.71 (1H, d, *J* =8.4, 6'-H), 7.71 (1H, d, *J*=8.4, 6'-H), 10.04 (1H, s, 4'-OH), 10.81 (1H, s, 7-OH). ^1H and ^{13}C NMR data were in agreement with the literature values of neoglycyrol [4].

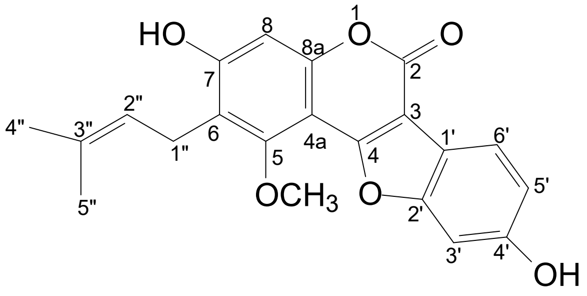

Neoglycyrol

GF-5: yellow needle crystal; HR-ESI/MS: *m/z* 269.0808 $[M+H]^+$; molecular formula: $C_{16}H_{12}O_4$; ^1H NMR (DMSO-d_6, δ, ppm): 3.77 (3H, s, 4'-OCH_3), 6.97 (2H, d, *J*=8.7, 3'-H), 7.49 (2H, d, *J*=8.7, 2'-H), 8.31 (1H, s, 2-H), 7.96 (1H, d, *J*=8.8, 5-H)，6.92 (1H, dd, *J*=8.8, 2.0, 6-H), 6.87 (1H, d, *J*=2.0, 8-H), 10.75 (1H, s, 7-OH); ^1H and ^{13}C NMR data were in agreement with the literature values of formononetin [5].

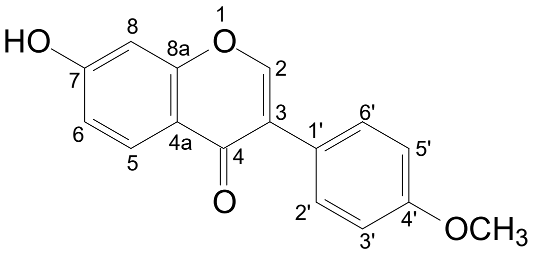

Formononetin

GF-6: yellow needle crystal; HR-ESI/MS: *m/z* 339.1590 $[M+H]^+$; molecular formula: $C_{21}H_{22}O_4$; ^1H

NMR (DMSO-d_6, δ, ppm): 1.45 (6H, s, 4"-H and 5"-H), 3.83 (3H, s, 2-OCH_3), 4.95 (1H, d, *J*=17.8, 3"a-H), 4.92 (1H, d, *J*=10.3, 3"b-H), 6.24 (1H, dd, *J*=17.8, 10.3, 2"-H), 6.52 (1H, s, 3-H), 6.88 (2H, d, *J*=8.5, 3'-H and 5'-H), 7.52 (1H, s, 6-H), 7.58 (1H, d, *J*=15.0, α-H), 7.89 (1H, d, *J*=15.0, β-H), 7.97 (2H, d, *J*=8.5, 2'-H and 6'-H), 10.28 (1H, s, 4'-OH), 10.10 (1H, s, 4-OH). ^{1}H and ^{13}C NMR data were identical with the literature values of licochalcone A [6].

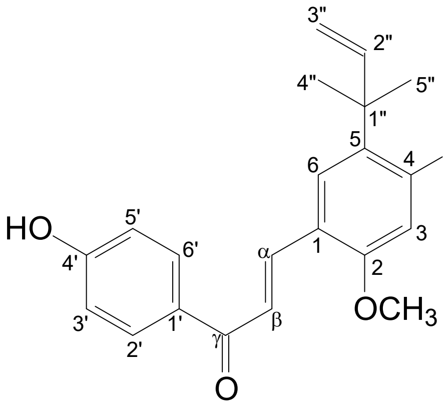

Licochalcone A

GF-7: white needle crystal; HR-ESI/MS: *m/z* 257.0809 $[M+H]^+$; molecular formula: $C_{15}H_{12}O_4$; ^{1}H NMR (DMSO-d_6, δ, ppm): 2.63 (1H, dd, *J*=16.8, 2.8, 3a-H), 3.10 (1H, dd, *J*=12.8, 16.7, 3b-H), 5.44 (1H, dd, *J*=12.8，2.7, 2-H), 6.33 (1H, d, *J*=2.1, 8-H)，6.50 (1H, dd, *J*=2.1, 8.6, 6-H), 6.79 (2H, d, *J*=8.5, 3'-H and 5'-H), 7.32 (2H, d, *J*=8.5, 2'-H and 6'-H), 7.64 (1H, d, *J*=8.7, 5-H), 10.51 (1H, s, 7-OH)，9.52 (1H, s, 4'-OH). ^{1}H and ^{13}C NMR data were identical with the literature values of liquiritigenin [5].

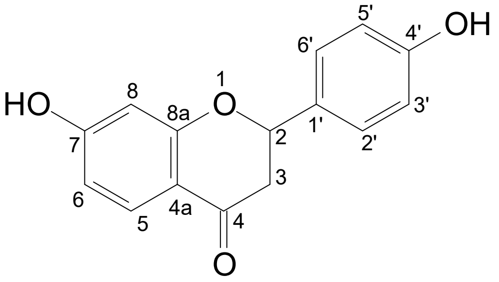

Liquiritigenin

GF-8: yellow needle crystal; HR-ESI/MS: *m/z* 257.0805 $[M+H]^+$; molecular formula: $C_{15}H_{12}O_4$; ^{1}H NMR (DMSO-d_6, δ, ppm): 6.42 (1H, d, *J*=8.6, 6'-H), 6.84 (2H, dd, *J*=8.6, 2.2, 5'-H), 7.51 (1H, d, *J*=15.0, α-H), 7.74 (2H, m, 3-H and 5-H)，7.72 (2H, m, 2-H, and 6-H), 7.82 (1H, d, *J*=15.0, β-H), 8.15 (1H, d, *J*=2.2, 3'-H), 10.10 (1H, s, 4-OH), 10.60 (1H, s,2'-OH), 13.57 (1H, s, 4'-OH). ^{1}H and ^{13}C NMR data were identical with the literature values of isoliquiritigenin [7].

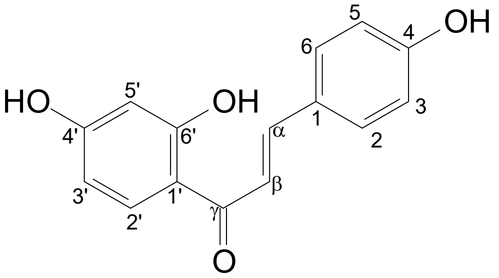

Isoliquiritigenin

GF-9: faint yellow needle crystal; HR-ESI/MS: *m/z* 323.1278 $[M+H]^+$; molecular formula: $C_{20}H_{18}O_4$; ^1H NMR (DMSO-d_6, δ, ppm): 1.68 (3H, s, 5''-H), 1.73 (3H, s, 4''-H), 3.29 (1H,d, *J*=7.3, 1''-H), 5.32 (1H, t, *J*=7.3, 2''-H), 6.69 (1H, s, 3-H), 6.92 (2H, d, *J*=8.7, 3'-H and 5'-H), 6.97 (1H, s, 8-H), 7.66 (1H, s, 5-H), 7.88 (2H, d, *J*=8.7, 2'-H and 6'-H), 10.21 (1H, s, 4'-OH), 10.81(1H, s, 7-OH). ^1H and ^{13}C NMR data were identical with the literature values of licoflavone [8].

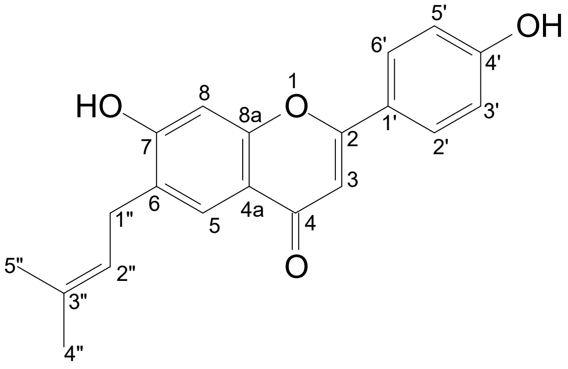

Licoflavone

GF-10: yellow needle crystal; HR-ESI/MS: *m/z* 315.0866 $[M+H]^+$; molecular formula: $C_{17}H_{14}O_6$; ^1H NMR (DMSO-d_6, δ, ppm): 3.86 (3H, s, 7-OCH_3), 3.80 (3H, s, 3-OCH_3), 6.36 (1H, d, *J*=1.8, 8-H), 6.72 (1H, d, *J*=1.8, 6-H), 6.95 (2H, d, *J*=8.7, 3'-H and 5'-H), 7.97 (2H, d, *J*=8.7, 2'-H and 6'-H), 10.25 (1H, s, 4'-OH), 12.66 (1H, s, 5-OH);. ^1H and ^{13}C NMR data were identical with the literature values of kumatakenin A [9].

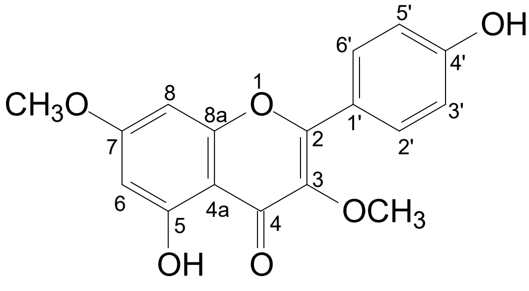

Kumatakenin A

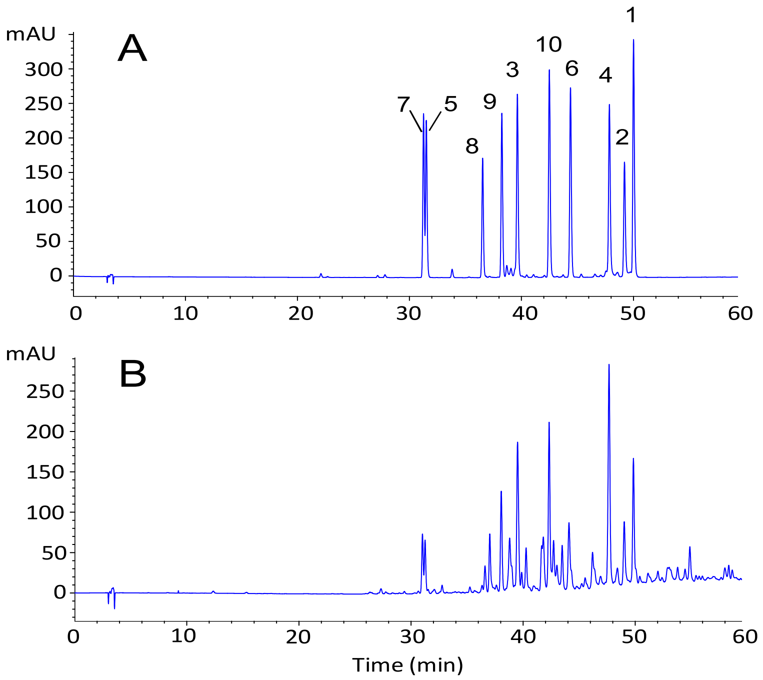

Part 2. HPLC of isolated flavonoids and dichloromethane extract of *G. uralensis*

(A) 10 flavonoids isolated from CH_2Cl_2 extract of *G. uralensis*; (B) CH_2Cl_2 extract of *G. uralensis*

References

1. Saitoh, T., Noguchi, H., & Shibata, S. A new isoflavone and the corresponding isoflavanone of licorice root. *Chemical and Pharmaceutical Bulletin*.1978, *26*, 144-147.

2. Liu, F., Ni, H., Qing, D. G., Meng, D. L., Li, N., & Jia, X. G. Chemical constituents from the residues of *Glycyrrhiza inflata* Batal. *Chinese Journal of Medicinal Chemistry*, 2011, *4*, 312-314.

3. Jayaprakasam, B., Doddaga, S., Wang, R., Holmes, D., Goldfarb, J., & Li, X. M. Licorice flavonoids inhibit eotaxin-1 secretion by human fetal lung fibroblasts in vitro. *Journal of agricultural and food chemistry*. 2009, *57*, 820-825.

4. Wang, C. L., Zhang, R. Y., Han, Y. S., Dong, X. G., & Liu, W. B. Chemical studies of coumarins from *Glycyrrhiza uralensis* Fisch. *Acta pharmaceutica Sinica*. 1991, *26*, 147-151.

5. Zhu, D. Y., Song, G. Q., Jiang, F. X., Chang, X. R., & Guo, W. B. Studies on chemical constituents of *Glycyrrhiza uralensis* Fisch---the structures of isolicoflavonol and glycycoumarin. *Acta Chimica Sinica*. 1984, *42*, 1080-1084.

6. Kajiyama, K., Demizu, S., Hiraga, Y., Kinoshita, K., Koyama, K., Takahashi, K., Kinoshita, T. Two prenylated retrochalcones from *Glycyrrhiza inflata. Phytochemistry*. 1992, *31*, 3229-3232.

7. Fukai, T., Marumo, A., Kaitou, K., Kanda, T., Terada, S., Nomura, T. Anti-Helicobacter pylori flavonoids from licorice extract. *Life sciences*. 2002, *71*, 1449-1463.

8. Yang, L., Che, Q. M., Bi, C., Sun, Q. S. Studies on flavonoids from the residues of *Glycyrrhiza inflata* Batal. *Chinese Traditional and Herbal Drugs*. 2007, *38*, 671-673.

9. Saitoh, T., Kinoshita, T., & Shibata, S. Flavonols of licorice root. *Chemical and Pharmaceutical Bulletin*. 1976, *24*, 1242-1245.
